# Effects of omega-3 fatty acid on major cardiovascular outcomes: A systematic review and meta-analysis

**DOI:** 10.1097/MD.0000000000029556

**Published:** 2022-07-29

**Authors:** Fangyu Yu, Shun Qi, Yanan Ji, Xizhi Wang, Shaohong Fang, Ruokui Cao

**Affiliations:** Taizhou Hospital of Traditional Chinese Medicine, Taizhou, Zhejiang Province, China.

**Keywords:** omega-3 fatty acid, cardiovascular disease, meta-analysis

## Abstract

**Background:**

The effects of omega-3 fatty acid on cardiovascular health obtained inconsistent results. A systematic review and meta-analysis were therefore conducted to assess the effects of omega-3 fatty acid supplementation for primary and secondary prevention strategies of major cardiovascular outcomes.

**Methods:**

The databases of PubMed, Embase, and the Cochrane library were systematically searched from their inception until September 2020. Relative risks (RRs) with 95% confidence intervals were used to assess effect estimates by using the random-effects model.

**Results:**

Twenty-eight randomized controlled trials involving 136,965 individuals were selected for the final meta-analysis. Omega-3 fatty acid was noted to be associated with a lower risk of major cardiovascular events (RR, 0.94; 95% CI, 0.89–1.00; *P* = .049) and cardiac death (RR, 0.92; 95% CI, 0.85–0.99; *P* = .022). However, no significant differences was noted between omega-3 fatty acid and the control for the risks of all-cause mortality (RR, 0.97; 95% CI, 0.92–1.03; *P* = .301), myocardial infarction (RR, 0.90; 95% CI, 0.80–1.01; *P* = .077), and stroke (RR, 1.02; 95% CI, 0.94–1.11; *P* = .694).

**Conclusions:**

Major cardiovascular events and cardiac death risks could be avoided with the use of omega-3 fatty acid. However, it has no significant effects on the risk of all-cause mortality, myocardial infarction, and stroke.

## 1. Introduction

Cardiovascular disease (CVD) is the leading cause of death accounting for 179 million deaths annually worldwide. The incidence of CVD remains high although patients at high cardiovascular risk were treated with primary and secondary prevention strategies.^[1–3]^ Patients still suffer substantial residual cardiovascular risk even if the CVD risk was significantly reduced in patients using appropriate treatment with statins.^[4]^ An elevated triglyceride level was considered as an independent factor for the high residual risk on subsequent CVD.^[5,6]^ Therefore, additional strategies should be applied to further reduce residual risk in patients.

Omega-3 fatty acids have already been approved by the US Food and Drug Administration to further reduce elevated triglyceride levels. However, studies found that long-chain omega-3 fatty acids, which including eicosapentaenoic (EPA) and docosahexaenoic acids (DHA), did not show CVD benefits, irrespective of primary or secondary prevention.^[7,8]^ Moreover, the use of omega-3 fatty acid showed better tolerability and safety for preventing further CVD risk.^[9]^ Furthermore, lowering of blood pressure, increasing plaque stability, and improving endothelial function are the potential benefits of omega-3 fatty acids.^[10–12]^ Furthermore, the effects of omega-3 fatty acids on the risk of major cardiovascular outcomes obtained inconsistent results. Numerous randomized controlled trials (RCTs) have already been completed. Khan conducted a systematic review and found EPA and DHA reduced cardiovascular mortality and improved cardiovascular outcomes.^[13]^ However, other omega-3 fatty acid (e.g., fish oils and α-linolenic acid) were not included in Khan’s study which also suggested favorable effect to cardiovascular outcomes.^[26]^ Therefore, these data should be entered into the meta-analysis and the pooled conclusions updated. Therefore, a systematic review and meta-analysis of RCTs were conducted to evaluate the effects of omega-3 fatty acid supplementation on major cardiovascular outcomes. Moreover, the effects of omega-3 fatty acid according to the different characteristics of patients were also illustrated.

## 2. Methods

### 2.1. Ethical approvement and clinical registration

This study is a meta-analysis and does not contain any information of patients and ethical approvement and clinical registration are not applicable.

### 2.2. Data sources, search strategy, and selection criteria

The Preferred Reporting Items for Systematic Reviews and Meta-Analysis Statement was used to guide the performance and conduct of this systematic review and meta-analysis.^[14]^ Included in this study were RCTs that investigated the effects of omega-3 fatty acid supplementation on major cardiovascular outcomes. However, the language of publication was restricted to English. The electronic databases of PubMed, Embase, and the Cochrane library were systematically searched for eligible studies using the following search terms: “omega-3 FA,” “omega-3 polyunsaturated fat,” “fish oils,” “ω-3 FA,” and “randomized controlled trial.” The publication data for the trials were from their inception until September 2020. The ongoing RCTs were also identified in https://clinicaltrials.gov/ which summarizes the trials that have already registered or have been completed but not yet published. The bibliographies of the retrieved trials were also manually reviewed for any new relevant trials.

Two reviewers independently performed the literature search and study selection. Inconsistencies between reviewers were resolved by group discussion. The trial was included if they met the following inclusion criteria: (1) participants (patients with cardiovascular disease (CVD) history or at high risk for CVD); (2) intervention (omega-3 fatty acid supplementation); (3) control (omega-6 fatty acid supplementation, placebo, or usual care); (4) outcome (the study should have reported at least one of the major cardiovascular events (MACEs), all-cause mortality, cardiac death, myocardial infarction (MI), and stroke); and (5) study design (the study had to have the RCT design).

### 2.3. Data collection and quality assessment

The data from the retrieved trials were independently abstracted by two reviewers. The collected data included the first author or the name of the study group, publication year, country, sample size, mean age, male gender (in percent), body mass index (BMI), smoking (in percent), hypertension (in percent), diabetes mellitus (DM), prevention, intervention, follow-up duration, and reported outcomes. The Jadad scale, which was based on randomization, concealment of the treatment allocation, blinding, completeness of follow-up, or the use of the intention-to-treat analysis, was used by two reviewers to independently assess the quality of the individual trial. The scale system ranged from 0–5.^[15]^ Conflicts on data collection and quality assessment between reviewers were settled by an additional reviewer who referred to the original article.

### 2.4. Statistical analysis

The results of MACEs, all-cause mortality, cardiac death, MI, and stroke in each trial were assigned as dichotomous data. In addition, the individual relative risk (RR) with 95% confidence interval (CI) was calculated before data pooling. Furthermore, random-effects were applied to calculate the pooled effect estimates considering the underlying variations across the included trials.^[16,17]^ The *I*^2^ and *Q* statistics were used to assess the heterogeneity across the included trials. Significant heterogeneity was defined as *I*^2^ > 50.0% or *P* < .10.^[18,19]^ Sensitivity analysis was conducted to assess the stability of pooled conclusions by sequentially excluding individual trials.^[20]^ Subgroup analyses were performed for MACEs, all-cause mortality, cardiac death, MI, and stroke according to sample size, mean age, male (in percent), BMI (in percent), smoking (in percent), hypertension (in percent), DM (in percent), prevention, follow-up, or study quality. Moreover, the interaction tests, which was based on Student’s *t*-distribution, was used to evaluate the differences between subgroups.^[21]^ The qualitative (funnel plot) and quantitative methods (Egger and Begg tests) were also used to evaluate reported outcomes of publication biases.^[22,23]^ The inspective level for pooled results is two-sided, and 0.05 was regarded as the cutoff. All statistical analyses in this study were conducted using the software STATA (version 10.0 StataCorp, College Station, TX).

## 3. Results

### 3.1. Search for published literature

**Figure 1.**
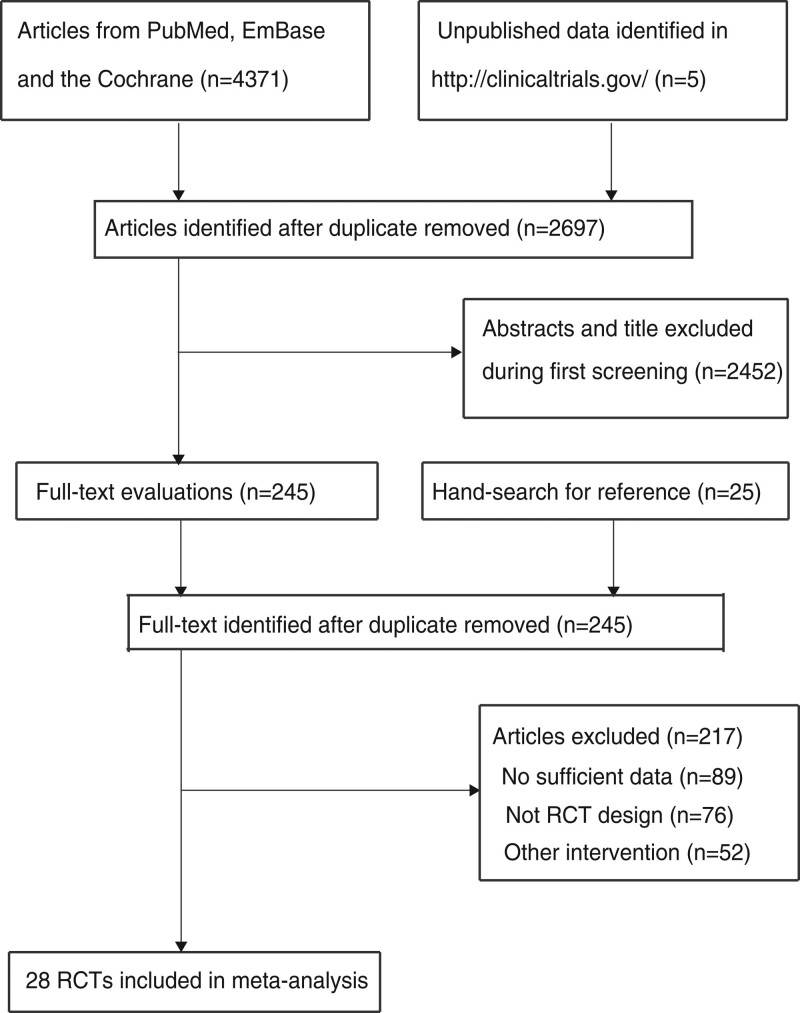
PRISMA flowchart for the literature search and trial selection.

Initial electronic searches identified 4371 records, and 2697 articles were retained after the duplicates were removed. Identified for full-text evaluations were 245 articles, and 217 studies were excluded because of insufficient data (n = 89), absence of an RCT design (n = 76), and other intervention (n = 52). Reviewing the reference lists of the remaining trials yielded 25 potentially eligible trials. All of these trials were included in initial electronic searches. The remaining 28 RCTs were then selected for the final meta-analysis ^[24–51]^. The details of the study selection are shown in Figure 1.

### 3.2. Characteristics of the included studies

Table 1 shows the baseline characteristics of the included studies and involved patients. Of the 28 included trials, 136,965 patients at high cardiovascular risk were recruited. The included trials were published between 1989 and 2019, and 101–25,871 patients were included in individual trials. Twelve and 18 trials applied omega-3 fatty acids as primary and secondary preventions, respectively. The mean follow-up duration ranged from 1–7.4 years, and the Jadad scale for the included trials ranged from 3–5. Twelve, ten, and six trials scored 5, 4, and 3, respectively. The trials that scored 4 or 5 in this study were considered as high quality.

**Table 1:** The summary characteristics in eligible study and involved individuals.

Study	Country	Sample size	Mean age (yr)	Male (%)	BMI (kg/m2)	Smoking (%)	Hypertension (%)	DM (%)	Prevention	Intervention	Follow-up	Study quality
Burr 1989 ^[23]^	UK	2033 (1015/1018)	56.5	100.0	NA	62.0	23.6	NA	Secondary	N-3 EPA + DHA vs nil or oily fish advice (or capsule) vs not	2.0 yr	3
Eritsland 1996 ^[24]^	Norway	610 (317/293)	60.0	86.9	25.3	19.2	22.3	6.9	Secondary	N-3 EPA + DHA vs nil	1.0 yr	4
GISSI-P 1999 ^[25]^	Italy	11324 (5666/5658)	59.4	85.3	26.5	42.4	35.6	14.8	Secondary	N-3 EPA + DHA vs nil	3.5 yr	5
Nilsen 2001 ^[26]^	Norway	300 (150/150)	64.0	79.3	26.0	38.7	24.3	10.3	Secondary	N-3 EPA + DHA vs corn oil	2.0 yr	3
Bemelmans 2002 ^[27]^	Netherlands	266 (109/157)	54.1	44.0	NA	49.2	48.5	NA	Primary	a-linolenic acid vs omega-6	2.0 yr	4
Burr 2003 ^[28]^	UK	3114 (1571/1543)	61.1	100.0	28.2	23.7	48.0	12.4	Secondary	Oily fish or capsules n-3 EPA + DHA vs nil	3.0–9.0 yr	3
Leaf 2005 ^[29]^	USA	402 (200/202)	65.5	83.1	NA	12.2	NA	NA	Secondary	N-3 EPA + DHA vs MUFA	1.0 yr	4
Raitt 2005 ^[30]^	USA	200 (100/100)	62.5	86.0	NA	NA	50.5	23.5	Secondary	N-3 EPA + DHA vs MUFA	2.0 yr	4
Brouwer 2006 ^[31]^	Europe (8 countries)	546 (273/273)	61.5	84.1	26.9	12.3	50.7	15.9	Secondary	N-3 EPA + DHA vs MUFA and n6	1.0 yr	5
Yokoyama 2007 ^[32]^	Japan	18645 (9326/9319)	61.0	31.5	24.0	19.0	35.5	16.0	Primary and secondary	EPA capsule vs nil	5.0 yr	4
GISSI-HF 2008 ^[33]^	Italy	6975 (3494/3481)	67.0	78.3	27.0	14.2	54.6	28.3	Secondary	N-3 EPA + DHA vs MUFA	3.9 yr	5
Tuttle 2008 ^[34]^	USA	101 (51/50)	58.0	74.3	30.5	27.7	46.5	19.8	Secondary	EPA + DHA vs MUFA	2.0 yr	4
Quinn 2010 ^[35]^	USA	402 (238/164)	76.0	47.8	26.0	23.4	NA	NA	Primary	N-3 DHA vs n-6 LA	1.5 yr	5
Kromhout 2010 ^[36]^	Netherlands	4837 (2404/2433)	69.0	78.1	27.8	16.8	89.7	21.0	Secondary	N-3 EPA + DHA vs nil	3.3 yr	5
Einvik 2010 ^[37]^	Norway	563 (282/281)	70.1	100.0	26.5	34.0	28.0	14.5	Primary	N-3 DHA + EPA vs n-6 LA also dietary advice intervention	3.0 yr	4
Rauch 2010 ^[38]^	Germany	3818 (1925/1893)	64.0	74.4	27.5	36.7	66.5	27.0	Secondary	Omega-3 vs olive oil	1.0 yr	5
Galan 2010 ^[39]^	France	2501 (1253/1248)	60.6	79.4	27.2	10.9	NA	NA	Primary	N-3 omega-3 vs paraffin (non-fat), also B vitamin comparison	4.0 yr	5
ORIGIN 2012 ^[40]^	40 locations in Europe and the Americas	12536 (6281/6255)	63.5	65.0	29.8	12.3	79.5	NA	Primary	N-3 omega-3 vs MUFA	6.0 yr	5
Macchia 2013 ^[41]^	Argentina	586 (289/297)	66.1	54.8	NA	7.6	91.4	12.9	Secondary	N-3 EPA + DHA vs MUFA	1.0 yr	4
Risk & Prevention 2013 ^[42]^	Italy	12513 (6244/6269)	64.0	61.5	NA	21.8	84.6	59.9	Primary	N-3 omega-3 vs olive oil	5.0 yr	4
Nigam 2014 ^[43]^	Canada	316 (153/163)	61.0	66.8	29.0	NA	43.4	8.2	Secondary	N-3 EPA + DHA vs n-6	1.0 yr	3
AREDS2 2014 ^[44]^	USA	4203 (2147/2056)	74.3	43.2	NA	56.6	NA	13.0	Primary	N-3 EPA + DHA vs nil	5.0 yr	5
Doi 2014 ^[45]^	Japan	115 (57/58)	70.0	74.8	24.0	34.8	68.7	37.4	Secondary	N-3 EPA vs nil	1.0 yr	3
Alfaddagh 2017 ^[46]^	USA	240 (126/114)	63.0	85.0	30.7	NA	83.3	28.3	Secondary	N-3 omega-3 vs nil	2.5 yr	3
ASCEND 2018 ^[47]^	UK	15480 (7740/7740)	63.3	62.6	30.8	8.3	NA	100.0	Primary	N-3 EPA + DHA vs MUFA	7.4 yr	5
Pahor 2019 ^[48]^	USA	289 (148/141)	77.6	52.6	31.4	NA	69.2	23.5	Primary	N-3 vs PUFA plus or minus losartan	1.0 yr	4
Bhatt 2019 ^[49]^	11 Countries in Westernised, Eastern Europe, Asia Pacific	8179 (4089/4090)	64.0	71.2	30.8	NA	NA	58.5	Primary and secondary	N-3 omega-3 vs paraffin oil	4.9 yr	5
Manson 2019 ^[50]^	USA	25871 (12933/12938)	67.1	49.4	28.1	7.2	49.8	13.7	Primary	N-3 omega-3 vs MUFA	5.3 yr	5

### 3.3. Major cardiovascular events

**Figure 2.**
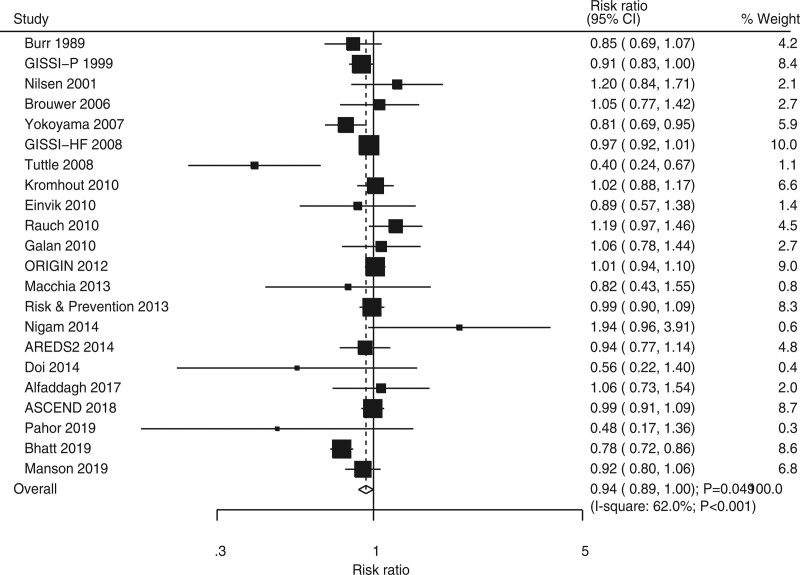
Forest plot for the effects of omega-3 fatty acids on the risk of major cardiovascular events.

Twenty-two RCTs showed the effect of omega-3 fatty acids on the risk of MACEs. Omega-3 fatty acids was associated with a reduced risk of MACEs (RR, 0.94; 95% CI, 0.89–1.00; *P* = .049; Fig. 2). In addition, significant heterogeneity was seen across included trials (*I*^2^ = 62.0%; *P* < 0.001). The pooled conclusion for MACEs was variable after sequentially excluding individual trials because of the marginal 95% CI (Supplemental Digital Content 1, http://links.lww.com/MD2/B85). Subgroup analysis suggested that the beneficial effect of omega-3 fatty acids on MACEs risk was mainly observed in the groups with a sample size of ≥1,000, a male proportion of ≥80.0%, omega-3 fatty acids used as primary prevention, follow-up duration of ≥3 years, and trials of high quality (Table 2). Moreover, the differences among subgroups based on smoking (*P* < .001) and hypertension proportions (*P* = .002) were associated with statistical significance. No significant publication bias for MACEs was observed (*P*-value for Egger, 0.648; *P* value for Begg, 0.236; Supplemental Digital Content 2, http://links.lww.com/MD2/B86).

**Table 2:** Subgroup analyses.

Outcomes	Variables	Group	RR and 95% CI	P value	Heterogeneity (%)	P value for heterogeneity	P value between subgroups
Major cardiovascular events	Sample size	= 1000	0.94 (0.89–1.00)	.038	65.3	.001	1.000
		< 1000	0.89 (0.68–1.17)	.406	61.4	.008	
	Mean age (yr)	= 60.0	0.96 (0.91–1.02)	.184	57.6	.001	.080
		< 60.0	0.76 (0.57–1.02)	.066	79.5	.008	
	Male proportion (%)	= 80.0	0.92 (0.85–0.99)	.036	0.0	.781	.399
		< 80.0	0.95 (0.88–1.01)	.122	69.7		
	BMI (kg/m^2^)	= 28.0	0.90 (0.79–1.04)	.158	81.6		.479
		< 28.0	0.97 (0.90–1.03)	.323	36.2	.119	
		Not reported	0.95 (0.89–1.04)	.295	0.0	.637	
	Smoking (%)	= 30.0	0.96 (0.86–1.07)	.479	34.4	.165	
		< 30.0	0.96 (0.91–1.02)	.161	50.0	.029	
		Not reported	0.96 (0.65–1.40)	.819	68.2	.024	
	Hypertension (%)	= 50.0	0.99 (0.95–1.02)	.486	0.0	.494	.002
		< 50.0	0.89 (0.78–1.01)	.076	63.0	.008	
		Not reported	0.92 (0.79–1.08)	.296	81.0	.001	
	DM (%)	= 20.0	0.96 (0.88–1.05)	.346	72.5		.134
		< 20.0	0.91 (0.81–1.02)	.108	54.9	.018	
		Not reported	0.99 (0.91–1.08)	.851	8.5	.335	
	Prevention	Primary	0.92 (0.85–1.00)	.050	68.0	.001	.237
		Secondary	0.97 (0.88–1.07)	.540	57.3	.007	
	Follow-up (yr)	= 3.0	0.94 (0.89–1.00)	.040	65.1	.001	.877
		< 3.0	0.94 (0.80–1.11)	.474	62.5	.003	
	Study quality	High	0.93 (0.88–1.00)	.037	70.0		.619
		Low	1.00 (0.86–1.15)	.949	28.4	.212	
All-cause mortality	Sample size	= 1000	0.98 (0.93–1.03)	.421	47.6	.029	.158
		< 1000	0.77 (0.56–1.07)	.121	7.7	.371	
	Mean age (yr)	= 60.0	0.99 (0.95–1.04)	.751	16.0	.255	.004
		< 60.0	0.79 (0.63–0.99)	.042	38.3	.182	
	Male proportion (%)	= 80.0	0.86 (0.70–1.05)	.135	61.0	.012	.155
		< 80.0	0.98 (0.94–1.02)	.358	4.7	.400	
	BMI (kg/m^2^)	= 28.0	0.99 (0.91–1.07)	.750	48.2	.085	.621
		< 28.0	0.97 (0.89–1.07)	.593	33.4	.123	
		Not reported	0.86 (0.67–1.11)	.258	41.9	.126	
	Smoking (%)	= 30.0	0.86 (0.71–1.04)	.130	38.9	.133	.017
		< 30.0	1.00 (0.95–1.04)	.919	12.0	.319	
		Not reported	0.73 (0.37–1.42)	.353	46.5	.171	
	Hypertension (%)	= 50.0	0.98 (0.92–1.04)	.504	13.6	.321	.763
		< 50.0	0.95 (0.83–1.09)	.492	61.8	.005	
		Not reported	0.94 (0.87–1.02)	.135	0.0	.667	
	DM (%)	= 20.0	0.96 (0.90–1.03)	.244	20.8	.265	.670
		< 20.0	0.99 (0.86–1.13)	.835	52.9	.024	
		Not reported	0.92 (0.79–1.09)	.334	28.5	.221	
	Prevention	Primary	0.99 (0.94–1.04)	.618	10.5	.346	.239
		Secondary	0.95 (0.85–1.06)	.336	46.5	.029	
	Follow-up (yr)	= 3.0	0.99 (0.94–1.04)	.682	27.7	.181	.024
		< 3.0	0.88 (0.73–1.06)	.178	28.6	.157	
	Study quality	High	0.97 (0.92–1.02)	.233	23.5	.171	.714
		Low	0.86 (0.61–1.23)	.415	66.8	.017	
Cardiac death	Sample size	= 1000	0.92 (0.85–1.00)	.050	48.7	.029	.205
		< 1000	0.70 (0.45–1.08)	.105	0.0	.702	
	Mean age (yr)	= 60.0	0.95 (0.88–1.02)	.146	20.6	.224	.015
		< 60.0	0.78 (0.67–0.92)	.003	9.2	.347	
	Male proportion (%)	= 80.0	0.83 (0.63–1.09)	.189	68.5	.004	.518
		< 80.0	0.93 (0.88–0.99)	.013	0.0	.768	
	BMI (kg/m^2^)	= 28.0	0.95 (0.82–1.10)	.492	64.8	.014	.431
		< 28.0	0.90 (0.84–0.97)	.007	0.0	.755	
		Not reported	0.85 (0.62–1.15)	.279	40.7	.150	
	Smoking (%)	= 30.0	0.80 (0.71–0.91)	.001	0.0	.721	.016
		< 30.0	0.97 (0.89–1.05)	.462	33.1	.134	
		Not reported	0.81 (0.67–0.98)	.032	0.0	.390	
	Hypertension (%)	= 50.0	0.95 (0.89–1.02)	.151	0.0	.594	.135
		< 50.0	0.91 (0.75–1.10)	.306	55.6	.021	
		Not reported	0.82 (0.72–0.94)	.004	0.0	.901	
	DM (%)	= 20.0	0.91 (0.85–0.97)	.006	0.0	.516	.774
		< 20.0	0.94 (0.76–1.15)	.518	52.3	.040	
		Not reported	0.86 (0.65–1.14)	.289	53.3	.092	
	Prevention	Primary	0.93 (0.86–1.00)	.053	0.0	.506	.838
		Secondary	0.91 (0.79–1.04)	.173	51.3	.025	
	Follow-up (yr)	= 3.0	0.95 (0.88–1.03)	.245	37.1	.112	.012
		< 3.0	0.80 (0.70–0.90)	< .001	0.0	.626	
	Study quality	High	0.92 (0.87–0.97)	.001	0.0	.708	.430
		Low	0.85 (0.52–1.37)	.500	80.7	.001	
Myocardial infarction	Sample size	= 1000	0.90 (0.79–1.02)	.091	63.4	.002	.818
		< 1000	0.97 (0.59–1.59)	.890	0.0	.431	
	Mean age (yr)	= 60.0	0.87 (0.77–0.99)	.028	47.9	.023	.101
		< 60.0	1.03 (0.68–1.55)	.889	46.8	.130	
	Male proportion (%)	= 80.0	1.07 (0.73–1.59)	.723	39.1	.177	.082
		< 80.0	0.87 (0.76–0.98)	.026	48.7	.021	
	BMI (kg/m^2^)	= 28.0	0.84 (0.68–1.04)	.102	79.5	.001	.424
		< 28.0	0.91 (0.79–1.04)	.165	0.6	.419	
		Not reported	1.01 (0.76–1.33)	.956	17.0	.304	
	Smoking (%)	= 30.0	1.09 (0.88–1.36)	.441	12.4	.336	.001
		< 30.0	0.89 (0.78–1.00)	.055	36.4	.117	
		Not reported	0.70 (0.60–0.82)		0.0	.515	
	Hypertension (%)	= 50.0	0.98 (0.87–1.11)	.762	2.4	.407	.051
		< 50.0	0.92 (0.72–1.17)	.501	58.1	.026	
		Not reported	0.85 (0.69–1.05)	.140	56.4	.076	
	DM (%)	= 20.0	0.82 (0.71–0.93)	.003	24.7	.249	
		< 20.0	0.83 (0.72–0.97)	.017	9.2	.359	
		Not reported	1.13 (0.97–1.31)	.127	3.9	.373	
	Prevention	Primary	0.86 (0.74–1.00)	.045	62.7	.006	.190
		Secondary	0.99 (0.80–1.23)	.948	21.0	.256	
	Follow-up (yr)	= 3.0	0.86 (0.75–0.98)	.022	61.3	.008	.053
		< 3.0	1.07 (0.83–1.38)	.588	10.2	.350	
	Study quality	High	0.86 (0.76–0.97)	.013	50.1	.020	.022
		Low	1.23 (0.92–1.64)	.167	0.4	.404	
Stroke	Sample size	= 1000	1.03 (0.93–1.13)	.616	32.7	.146	.861
		< 1000	0.92 (0.36–2.35)	.861	0.0	.736	
	Mean age (yr)	= 60.0	1.00 (0.92–1.09)	.976	10.7	.340	.171
		< 60.0	1.23 (0.92–1.64)	.163	0.0	.545	
	Male proportion (%)	= 80.0	1.23 (0.91–1.64)	.174	-	-	.183
		< 80.0	1.00 (0.92–1.09)	1.000	4.7	.400	
	BMI (kg/m^2^)	= 28.0	0.94 (0.84–1.05)	.279	18.0	.297	.047
		< 28.0	1.11 (0.97–1.27)	.145	0.0	.771	
		Not reported	1.23 (0.95–1.59)	.112	0.0	.710	
	Smoking (%)	= 30.0	1.17 (0.92–1.48)	.193	0.0	.671	.019
		< 30.0	1.03 (0.95–1.12)	.511	0.0	.663	
		Not reported	0.73 (0.56–0.94)	.015	0.0	.796	
	Hypertension (%)	= 50.0	1.08 (0.90–1.29)	.436	29.6	.224	.372
		< 50.0	1.07 (0.93–1.23)	.322	0.0	.758	
		Not reported	0.94 (0.77–1.14)	.512	41.4	.163	
	DM (%)	= 20.0	1.02 (0.82–1.28)	.851	62.5	.031	.354
		< 20.0	1.08 (0.95–1.23)	.252	0.0	.926	
		Not reported	0.94 (0.81–1.08)	.365	0.0	.695	
	Prevention	Primary	0.99 (0.89–1.09)	.795	23.5	.234	.063
		Secondary	1.19 (0.99–1.44)	.065	0.0	.916	
	Follow-up (yr)	= 3.0	1.01 (0.91–1.11)	.872	31.1	.169	.226
		< 3.0	1.19 (0.90–1.58)	.213	0.0	.803	
	Study quality	High	1.02 (0.93–1.12)	.684	23.8	.210	.757
		Low	1.08 (0.72–1.61)	.719	0.0	.645	

### 3.4. All-cause mortality

**Figure 3.**
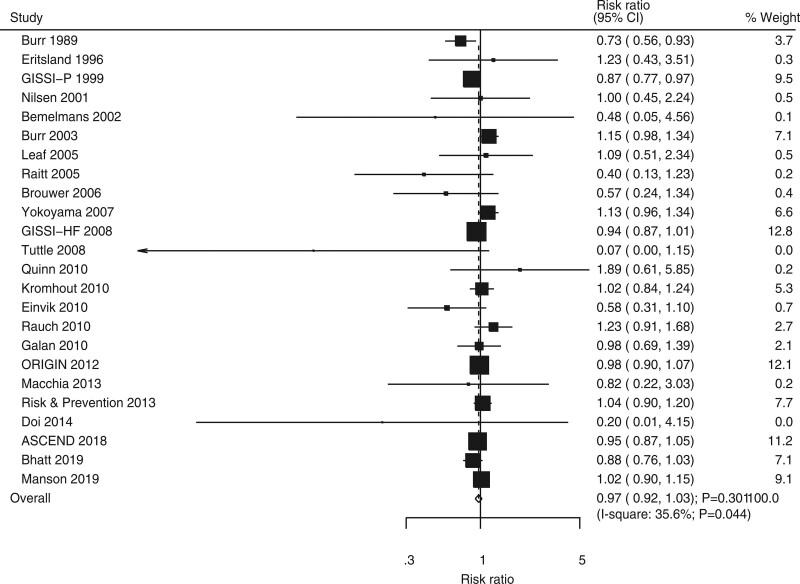
Forest plot for the effects of omega-3 fatty acids on the risk of all-cause mortality.

Twenty-four RCTs showed the effect of omega-3 fatty acids on the risk of all-cause mortality. No significant difference was noted between omega-3 fatty acids and control for the risks of all-cause mortality (RR, 0.97; 95% CI, 0.92–1.03; *P* = .301; Fig. 3). Potential significant heterogeneity was detected across included trials (*I*^2^ = 35.6%; *P* = .044). The pooled conclusion was robustness and was not changed when a sensitivity analysis was conducted (Supplemental Digital Content 1, http://links.lww.com/MD2/B85). Subgroup analysis suggested that omega-3 fatty acids could protect against all-cause mortality risk when the mean age of individuals was <60 years (Table 2). Moreover, the effects of omega-3 fatty acids on the risk of all-cause mortality could be affected by mean age (*P* = .004), smoking proportion (*P* = .017), and follow-up duration (*P* = .024). No significant publication bias was noted for all-cause mortality (*P* value for Egger, 0.337; *P* value for Begg, 0.309; Supplemental Digital Content 2, http://links.lww.com/MD2/B86).

### 3.5. Cardiac death

**Figure 4.**
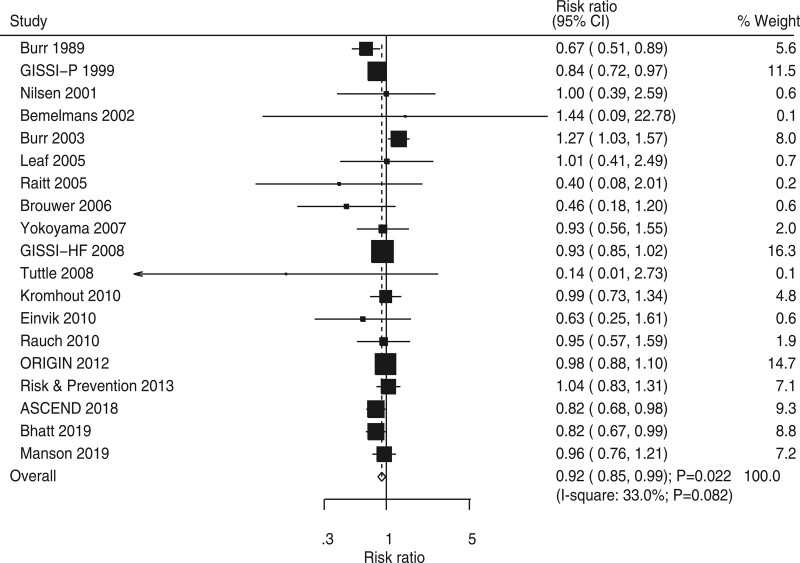
Forest plot for the effects of omega-3 fatty acids on the risk of cardiac death.

Nineteen RCTs showed the effect of omega-3 fatty acids on the risk of cardiac death. The pooled RR indicated that omega-3 fatty acids could protect against cardiac death risk (RR, 0.92; 95% CI, 0.85–0.99; *P* = .022; Fig. 4) and potential heterogeneity among included trials (*I*^2^ = 33.0%; *P* = .082). The pooled conclusion for cardiac death risk was variable owing to the marginal 95% CI (Supplemental Digital Content 1, http://links.lww.com/MD2/B85). Subgroup analysis found that the beneficial effects of omega-3 fatty acids on cardiac death were mainly observed in the groups with a sample size of ≥1,000, mean age of <60 years, a male proportion of <80%, BMI of <28 kg m^−2^, the smoking proportion of ≥30% or trials that did not report smoking proportion, trials that did not report hypertension proportion, DM proportion of ≥20%, follow-up duration of <3 years, and trials of high quality (Table 2). Moreover, the risk of cardiac death for the use of omega-3 fatty acids could be affected by mean age (*P* = .015), smoking proportion (*P* = .016), and follow-up duration (*P* = .012). Moreover, no significant publication bias for cardiac death was detected (*P* value for Egger, .282; *P* value for Begg, 0.576; Supplemental Digital Content 2, http://links.lww.com/MD2/B86).

### 3.6. Myocardial infarction

**Figure 5.**
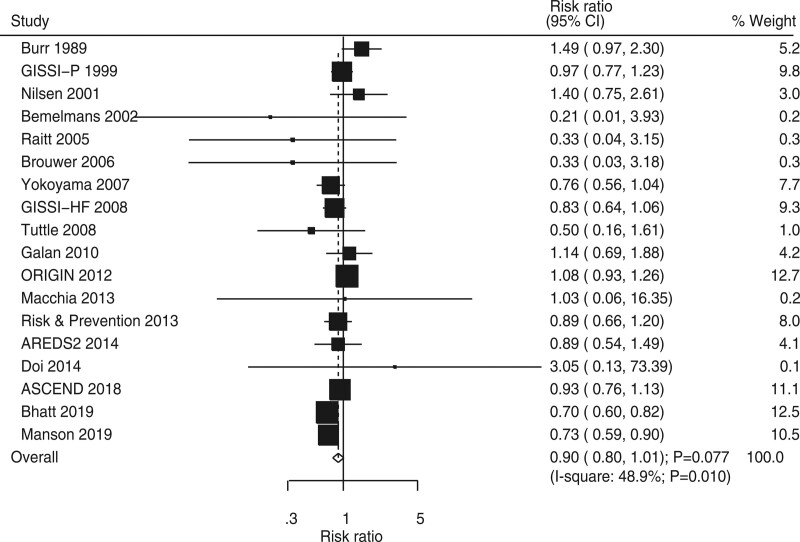
Forest plot for the effects of omega-3 fatty acids on the risk of myocardial infarction.

Eighteen RCTs showed the effect of omega-3 fatty acids on the risk of MI. Omega-3 fatty acids was noted to not be associated with a reduced risk of MI (RR, 0.90; 95% CI, 0.80–1.01; *P* = .077; Fig. 5), and significant heterogeneity was detected across included trials (*I*^2^ = 48.9%; *P* = .010). Sensitivity analysis indicated that the risk of MI may be reduced by sequentially excluding individual trials (Supplemental Digital Content 1, http://links.lww.com/MD2/B85). Subgroup analysis suggested that omega-3 fatty acids significantly reduced the risk of MI when the mean age was ≥60 years, the male proportion was <80%, trials on smoking proportion were not reported, DM proportion was ≥20% or <20%, omega-3 fatty acids were used as primary prevention, follow-up duration was ≥3 years, and trials were of high quality (Table 2). Moreover, smoking proportion (*P* = .001), DM proportion (*P* <.001), and study quality (*P* = .022) could affect the effects of omega-3 fatty acids on the risk of MI. No significant publication bias exists for the risk of MI (*P* value for Egger, .979; *P* value for Begg, .880; Supplemental Digital Content 2, http://links.lww.com/MD2/B86).

### 3.7. Stroke

**Figure 6.**
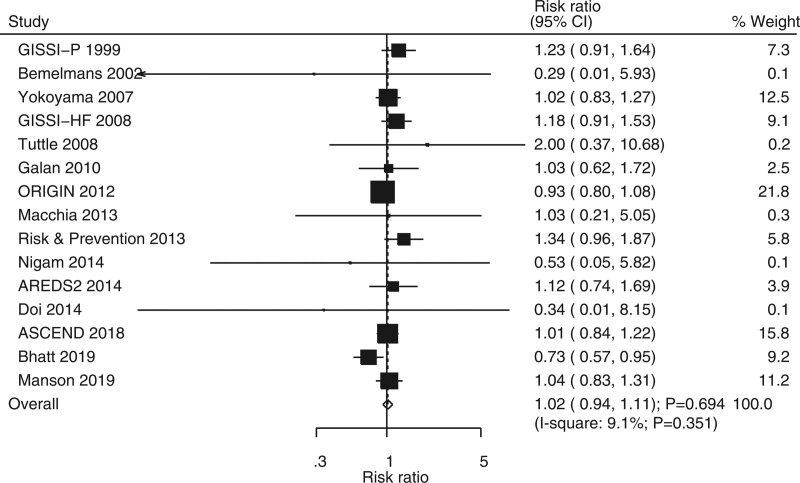
Forest plot for the effects of omega-3 fatty acids on the risk of stroke.

Fifteen RCTs showed the effect of omega-3 fatty acids on the risk of stroke. No significant differences were noted between omega-3 fatty acids and control for the risk of stroke (RR, 1.02; 95% CI, 0.94–1.11; *P* = .694; Fig. 6). In addition, unimportant heterogeneity was seen among the included trials (*I*^2^ = 9.1%; *P* = .351). The pooled conclusion was robustness and was not altered by sequentially excluding individual trials (Supplemental Digital Content 1, http://links.lww.com/MD2/B85). Subgroup analysis suggested that omega-3 fatty acids could protect against stroke risk when pooled trials did not report smoking proportion (Table 2). Moreover, the effects of omega-3 fatty acids on the risk of stroke could be affected by BMI (*P* = .047) and smoking proportion (*P* = .019). No significant publication bias was detected for the risk of stroke (*P* value for Egger, .893; *P* value for Begg, .767; Supplemental Digital Content 2, http://links.lww.com/MD2/B86).

## 4. Discussion

An observational study initially reported the potential role of omega-3 fatty acids for in preventing the risks of major cardiovascular outcomes.^[52]^ However, this effect lacks further intervention RCTs confirmed to date. The current study included RCTs and assessed the effects of omega-3 fatty acids on the outcomes of MACEs, all-cause mortality, cardiac death, MI, and stroke. This comprehensive, quantitative meta-analysis involved 136,965 individuals from 28 trials across a wide range of characteristics. Furthermore, this study suggested that omega-3 fatty acids could protect against the risk of MACEs and cardiac death. However, omega-3 fatty acids were not associated with the risk of all-cause mortality, MI, and stroke. The effects of omega-3 fatty acids could be affected by mean age, BMI, smoking proportion, hypertension proportion, DM proportion, follow-up duration, and study quality as found in the results of subgroup analysis.

The role of omega-3 fatty acids on major cardiovascular outcomes have already been illustrated in several systematic reviews and meta-analyses. A meta-analysis conducted by Marik et al contained 11 RCTs and found that dietary supplementation with omega-3 fatty acids could reduce the risk of nonfatal MACEs, cardiac death, sudden cardiac death, and all-cause mortality. Thus, it should be applied as a secondary prevention for major cardiovascular outcomes.^[53]^ On the one hand, Filion et al conducted a meta-analysis of 29 RCTs and found that omega-3 fatty acids did not yield significant benefits on the risk of all-cause mortality and restenosis for patients at high cardiovascular risk.^[54]^ On the other hand, Kwak et al performed a meta-analysis of 14 RCTs and found that the use of omega-3 fatty acids as secondary prevention did not contribute sufficient effects on MACEs for patients with CVD history.^[55]^ Moreover, a meta-analysis conducted by Rizos et al included 20 RCTs and found that the use of omega-3 fatty acids did not yield significant benefits for cardiovascular outcomes.^[56]^ Furthermore, Casula et al conducted a meta-analysis of 11 RCTs to assess the effects of long-term omega-3 fatty acids for the secondary prevention of major cardiovascular outcomes and found the protective role of long-term high-dose omega-3 fatty acids on the risk of cardiac death, sudden death, and MI for patients with CVD history.^[57]^ In addition, a meta-analysis conducted by Wen et al included 14 RCTs and found that omega-3 fatty acids have no significant effect on the risk of MACEs while it could reduce the risk of all-cause mortality, cardiac death, and sudden cardiac death for patients with coronary heart disease.^[58]^ Moreover, Aung et al conducted a meta-analysis of 10 RCTs and found that omega-3 fatty acids were not associated with the risk of fatal or nonfatal coronary heart disease or MACEs.^[59]^ Furthermore, Popoff et al conducted a meta-analysis of 10 RCTs and found that omega-3 fatty acids did not provide significant benefits on cardiovascular health for patients after acute MI.^[60]^ However, several new published RCTs should be included and the pooled conclusions needed to be updated. Therefore, the current systematic review and meta-analysis were conducted to assess the effects of omega-3 fatty acids on major cardiovascular outcomes.

In summary, the results suggested that omega-3 fatty acids could protect against the risk of MACEs. Most of the included trials did not find significant differences between omega-3 fatty acids and control, while four trials reported a similar conclusion.^[26,33,35,50]^ The GISSI-Prevenzione trial found that dietary supplementation with omega-3 fatty acids could yield significant benefits on MACEs (all-cause mortality, nonfatal MI, and nonfatal stroke).^[26]^ The Japan EPA Lipid Intervention Study trial suggested that the use of eicosapentaenoic acid should be considered as a promising strategy for the prevention of MACEs for hypercholesterolemic patients.^[33]^ The THIS-DIET trial found active intervention with the Mediterranean-style diet and could provide significant benefits on cardiovascular health in patients after MI.^[35]^ The REDUCE-IT trial found that the risk for MACEs was significantly reduced for patients with elevated triglyceride levels applied with 2 g of omega-3 fatty acids.^[50]^ The potential reason for this could be that omega-3 fatty acids have antiarrhythmic effects.^[61,62]^ Moreover, the use of omega-3 fatty acids could reduce platelet aggregation,^[63,64]^ vasodilation,^[65,66]^ antiproliferation,^[67]^ plaque stabilization,^[68]^ and reduction in lipid action.^[69,70]^

The use of omega-3 fatty acids was noted to prevent the risk of cardiac death. However, it has no significant effects on the risk of all-cause mortality, MI, and stroke. The protective role of omega-3 fatty acids on cardiac death could be explained by the low dose of omega-3 fatty acids that could prevent sudden cardiac death through an antiarrhythmic effect.^[71]^ Sensitivity analysis found that omega-3 fatty acids may play a beneficial effect on the risk of all-cause mortality. This result could be explained by the high proportion of death caused by cardiac reasons. Furthermore, the use of omega-3 fatty acids did not affect the risk of MI and stroke. These results could be affected by the dose and duration of omega-3 fatty acid supplementation.

Significant heterogeneity exists for several major cardiovascular outcomes, and subgroup analysis was performed to assess the role of omega-3 fatty acids in patients with specific characteristics. Mean age, BMI, smoking proportion, hypertension proportion, DM proportion, follow-up duration, and study quality were noted to affect the effects of omega-3 fatty acids on major cardiovascular outcomes. Several reasons could explain these results. First, cardiovascular risk could be affected by the mean age of the patients, and the proportion of comorbidity across patients is different, which could affect the progression of major cardiovascular outcomes. Second, the role of omega-3 fatty acids may be more evident for patients at low cardiovascular risk, including the characteristics of BMI, smoking, hypertension, and DM proportion. (3) Third, the follow-up duration is significantly correlated with the duration of the use of omega-3 fatty acids and the events of interest outcome. (4) Lastly, the quality of the trials was related to the evidence level and the reliability of the pooled conclusions.

Several limitations of this study should be mentioned. First, the type of omega-3 fatty acids may affect the progression of major cardiovascular outcomes. Second, the treatment effect between the omega-3 fatty acids and control could be affected by the background intake of omega-3 fatty acids and other treatment strategies. Third, the definition of MACEs is different across the included trials, and the risk of MACEs for individuals using omega-3 fatty acids could be affected. Fourth, the subgroup analyses according to background therapies were not conducted because the stratified data according to the specific treatment strategy were not available. Lastly, inherent limitations exist for meta-analysis based on pooled data, including inevitable publication bias and restricted detailed analyses.

In conclusion, this study found that the use of omega-3 fatty acids could significantly reduce the risk of MACEs and cardiac death. However, no significant differences were found between omega-3 fatty acids and control for the risk of all-cause mortality, MI, and stroke. Further large-scale RCT should be conducted to assess the effects of omega-3 fatty acids on major cardiovascular outcomes. In addition, a cumulative meta-analysis should be conducted to assess the pooled effect estimates in clinical practice.

## Author contributions

Conceptualization: Fangyu Yu, Shun Qi.

Data curation: Ruokui Cao, Shaohong Fang, Shun Qi, Xizhi Wang, Yanan Ji.

Formal analysis: Fangyu Yu, Yanan Ji.

Methodology: Fangyu Yu.

Project administration: Fangyu Yu.

Writing – original draft: Fangyu Yu, Ruokui Cao, Shaohong Fang, Shun Qi, Xizhi Wang, Yanan Ji.

Writing – review & editing: Ruokui Cao, Shaohong Fang, Shun Qi, Xizhi Wang.
